# Spinal anaesthesia at low and moderately high altitudes: a comparison of anaesthetic parameters and hemodynamic changes

**DOI:** 10.1186/s12871-015-0104-y

**Published:** 2015-09-10

**Authors:** Mehmet Aksoy, Ilker Ince, Ali Ahıskalıoglu, Omer Karaca, Fikret Bayar, Ali Fuat Erdem

**Affiliations:** 1 Department of Anaesthesiology and Reanimation, Faculty of Medicine, Ataturk University, Erzurum, Turkey 25240; 2 Department of Anaesthesiology and Reanimation, Ordu State Hospital, Ordu, Turkey; 3 Department of Anaesthesiology and Reanimation, Faculty of Medicine, Sakarya University, Sakarya, Turkey

## Abstract

**Background:**

Hypoxemia caused high altitude leads to an increase and variability in CSF volume. The purpose of this prospective study was to detect the differences, if any, between moderately highlanders and lowlanders in terms of anaesthetic parameters under neuroaxial anaesthesia.

**Methods:**

Consecutive patients living at moderately high altitude (Erzurum, 1890 m above the sea level) and sea level (Sakarya, 31 m above the sea level) scheduled for elective lower extremity surgery with spinal anaesthesia were enrolled in this study (*n* = 70, for each group). Same anaesthesia protocol was applied for all patients. Spinal anaesthesia was provided with hyperbaric bupivacaine 0.5 %, 9 mg (1.8 mL) in all patients. Anaesthetic characteristics and hemodynamic parameters of patients were recorded. The findings obtained in two different altitudes were compared using appropriate statistical tests. If data was not normally distributed, comparisons were determined using the Mann–Whitney *U*-test. Comparisons were determined using the Independent *T* test when data was normally distributed and Fisher’s exact test was used to compare the percentage values.

**Results:**

Duration of the block procedure (minutes) was significantly shorter at the sea level (14.34 ± 0.88) than at moderate altitude (20.38 ± 1.46) (*P* < 0.001). Motor block duration (minutes) was higher at the sea level compared to the moderate altitude (310.2 ± 104.2, 200.4 ± 103.2; respectively; *P* < 0.05). Also, the sensory block time (minutes) was higher at the sea level compared to moderate altitude (200.2 ± 50. minutes vs. 155.2 ± 60.7 min; respectively; *P* < 0.05). Moderate altitude group had significantly higher MABP values at baseline, during surgery and at postoperative 1^st^ and 2nd hours than in the sea level group (*P* < 0.05, for all). Moderately high altitude group had lower heart rate values at baseline, during surgery and postoperative 1^st^ and 2^nd^ hours compared with the sea level group (*P* < 0.05). PDPH was seen more frequently (7.14 vs. 2.85 %; *P* < 0.05) at moderate altitude.

**Conclusions:**

Hemodynamic variations and more anaesthetic requirements following the spinal anaesthesia may be observed at moderately high altitudes compared to the sea level.

**Trial registration:**

Australian New Zealand Clinical Trials Registry: ACTRN12614000749606.

## Background

Individuals living at high and moderate altitudes have respiratory, cardiovascular and haematological changes in relation to the oxygen uptake and transport [1, 2]. An increase in blood viscosity, a decrease in carbon monoxide diffusion capacity, an increase in cerebral arterial blood flow, a reduction in blood volume and a decline in cardiac output occur in response to hypoxemia at high altitude [2]. It has been reported that the amount of cerebrospinal fluid (CSF) increases [3] and acid–base balance of CSF changes because of hypoxemia at high altitude [4]. On the other hand, Sorensen et al. [5] showed lower pH values of CSF in high-landers than in low-landers. They suggested that these differences in pH values of CSF may be associated with hyperventilation in high-altitude natives.

Spinal anaesthesia provides nerve blockade in a large part of the body during surgery with a smaller dose of local anaesthetic and shorter surgery onset time. However, it is difficult to keep the spread of local anaesthetic under control through the CSF after adequate block for surgery was provided [6]. So, there is an increased risk of complications due to extensive spread of local anaesthetic through the CSF. Many factors such as age, height, weight and the lumbosacral cerebrospinal fluid volume affect the intrathecal spread of the injected local anaesthetics [6]. It was shown that hypoxemia caused high altitude leads to an increase and variability in CSF volume [3] and these changes are the most important factor that contributes to the variability in the spread of the spinal sensory anaesthesia [7].

The high altitude anaesthesia literature is limited [8, 9] and the effect of moderately high altitude on central neuraxial block has not been studied. Because there is clinical evidence demonstrating the changes in CSF volume and contents under hypoxic conditions [3–5], we hypothesized that duration of sensory and motor blockade in lowlanders would be equal or greater than that in moderately highlanders. Therefore, the purpose of this prospective study was to detect the differences, if any, between moderately highlanders and lowlanders in terms of anaesthetic parameters under neuroaxial anaesthesia.

## Methods

This prospective study was approved by the Ethics Committee of Ataturk University, Medical Faculty, Erzurum, Turkey (the date of approval by the ethics committee: 26.12.2013, the protocol number: 12). A total of 155 consecutive male subjects who were admitted to 2 study institutions at Ataturk University, Medical Faculty, Erzurum, Turkey (1890 m above the sea level) and Sakarya University, Medical Faculty, Sakarya, Turkey (31 m above the sea level) between January 1, 2014 and June 30, 2014 and who were scheduled elective lower extremity surgery with spinal anaesthesia were enrolled in this study. Patients with the age between 25 and 40 years, a body mass index between 20 and 25 kg/m2, ASA (the classification of the American Society of Anesthesiologists) physical status I or II were included [8]. Smokers, alcohol consumers, patients with psychiatric or neurological disorders, chronic diseases such as diabetes, a body mass index over 25, ASA physical status III or IV and contraindications to spinal anaesthesia such as coagulation disorder and infection at the puncture site were excluded from the study. All participants were permanently resident at moderately high altitude, as well as the sea level. Written informed consent was obtained from all participating patients. Demographic characteristics (age, weight and height) and indications for surgery of the patients were recorded. Before transferred to the operating room, every patient received an infusion of 500 mL ringer’s lactate solution in 30 min via 18-gauge cannula in a forearm peripheral vein. Standard monitoring included non-invasive arterial pressure, electrocardiography and pulse oximetry were established for all patients in the operating room and post-anaesthesia care unit (PACU). Same anaesthesia protocol was applied for the patients living at moderately high altitude and the sea level. Each patient was premedicated with intravenous (iv) fentanyl (0.1 μg/kg) and midazolam (2 mg). After the skin infiltration with 2 % lidocaine, 26-gauge Quincke’s needle was inserted through the L_2–3_/ L_3–4_ intervertebral space of patient in sitting position. Once free flow of the cerebrospinal fluid was obtained, hyperbaric bupivacaine 0.5 %, 9 mg (1.8 mL) was injected intrathecally. Then, the patient was enrolled in the supine position. Sensory block level was tested using pinprick tests and motor block level was evaluated with Modified Bromage scale (scale 0 = full flexion of foot, knee and hip, ie, no motor block; scale 1 = full flexion of foot and knee, unable to hip flexion; scale 2 = full flexion of foot, unable to knee and hip flexion; scale 3 = total motor block; unable to foot, knee, and hip flexion). When the sensory block reached the T_12_ dermatome, surgery was initiated. If no signs of analgesia were observed within the first 10 min after the intrathecal injection, technique was considered as failed and general anaesthesia was administered for these patients. Oxygen was delivered with a face mask during surgery; iv midazolam (1 mg) for the complaint of discomfort was administered to each patient if necessary. During the operation, patients’ mean arterial blood pressure (MABP), heart rate (HR) were monitored and recorded every 5 min. Ephedrine (iv, 2.5 mg) was administered in case of hypotension (a 30 % decrease in systolic blood pressure compared to preoperative values) and atropine (iv, 0.5 mg) was applied when bradycardia (the heart rate < 45 beats/min) was observed. The application time of the spinal anaesthesia, duration of the block procedure (the time from the start of the anaesthetic procedure to the development of full motor block), duration of surgery (the time from the start of the surgical incision to the completion of surgery), highest sensory block level, anaesthetic complications and the number of patients required midazolam during the surgical procedure were recorded. After the surgery, patients were transferred to the PACU. In PACU, postoperative analgesia was provided with iv 50 mg tramadol and an anaesthetist assessed the sensory and motor block levels every half-hour from the end of surgery until there is a complete recovery of the motor block and recovery of the sensation of the S_2_ dermatome. Sensory block time (from the local anaesthetic injection to the recovery of S_2_ dermatome), motor block duration (the time from the local anaesthetic injection to the complete motor function recovery) were recorded. Following a complete recovery of the motor and sensory blocks, patients were transferred to the orthopaedic ward. On the first and seventh days after the operation, patients were questioned in terms of post-dural puncture headache (PDPH, increased pain intensity upon standing up from a supine position) by an investigator via telephone interview. Crystalloid infusions (500 ml, 8-h intervals) and a non-steroidal anti-inflammatory drug applied to the patients diagnosed with PDPH.

Sample size was calculated as minimum 63 patients, based on our preliminary results to detect a minimum difference of 25 % in the duration of sensory block between the two groups with a power of 80 %, α of 0.05 and β of 0.20 [10]. Data were analysed using SPSS software 12.0 (SPSS Inc., Chicago, IL, USA) and calculated as mean ± standard deviation, *P* < 0.05 was considered significant. The findings obtained in two different altitudes were compared using appropriate statistical tests. The Kolmogorov-Smirnov test was used to assess the normal distribution of data. If data was not normally distributed, comparisons were determined using the Mann–Whitney *U*-test. Comparisons were determined using the Independent *T* test when data was normally distributed and Fisher’s exact test was used to compare the percentage values.

## Results

**Fig. 1:**
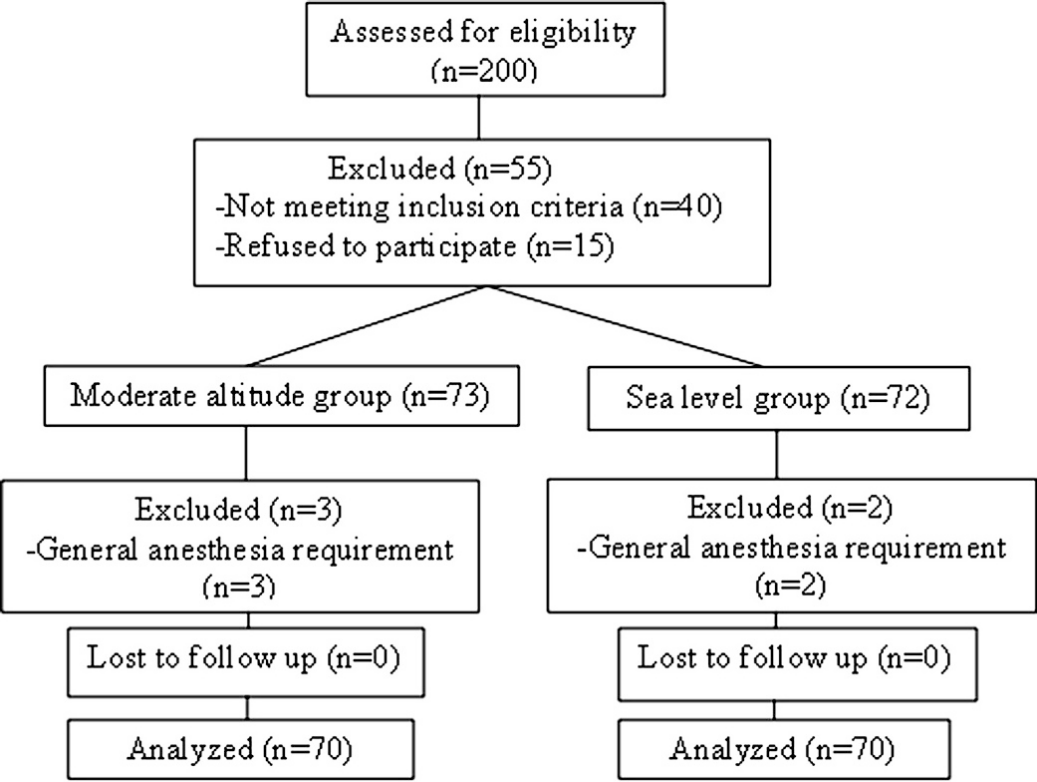
CONSORT flow diagram. The course of patients through this study was shown

Eligible patients for this study were analysed for the primary outcomes and are shown in the CONSORT flow diagram (Fig. 1) [11]. During study period, 200 patients were eligible for this study in two institutes and 160 patients had inclusion criteria. One hundred and forty five patients agreed to participate in the study. Five patients were excluded from the study due to general anaesthesia requirement caused by prolonged surgery. Study population was consisted of 140 patients divided as 70 patients in each group. Clinical characteristics of the patients in both groups were similar. However, moderate altitude group had higher baseline MABP and lower baseline heart rate values than in the sea level group (*P* < 0.05) (Table 1). The spinal anaesthesia was performed at the L_3–4_ (*n* = 120), L_2–3_ (*n* = 20, nine in moderately high altitude group and eleven in the sea level group) interspace at the midline. Adequate surgical anaesthesia was provided within 15 min in all patients. The application time of the anaesthetic technique (5.85 ± 0.75 min vs. 5.90 ± 1.65 min) and duration of the surgery (52.15 ± 20.25 min vs. 51.68 ± 15.19 min) were similar between the groups. Duration of the block procedure was significantly shorter at the sea level (14.34 ± 0.88 min) than at moderate altitude (20.38 ± 1.46 min; *P* < 0.001). No patient in both groups required midazolam during surgery. Eight patients in the sea level group and 3 patients in the moderately high altitude group needed ephedrine 5–30 mg for treatment of hypotension during surgery (*P* < 0.05). PDPH was seen more frequently (7.14 vs. 2.85 %; *P* < 0.05) at moderate altitude (Table 2). Motor block duration (minutes) was higher at the sea level compared to moderate altitude (310.2 ± 104.2, 200.4 ± 103.2; respectively; *P* < 0.05). Also, the sensory block time (minutes) was higher at the sea level compared to moderate altitude (200.2 ± 50. minutes vs. 155.2 ± 60.7 min; respectively; *P* < 0.05) (Table 2). The upper limit of the sensory blockade was significantly higher at the sea level compared to moderately high altitude (T_8_ vs. T_10_) (*P* < 0.001) (Table 3). The period from the local anaesthetic injection to the complete motor function recovery was shorter in moderately high altitude group than in the sea level group (Table 4).

**Table 1:** Baseline characteristics of the patients living at the sea level and moderately high altitude

	Moderately high altitude group	Sea level group
	(*n* = 70)	(*n* = 70)
Age (years)	41.61 ± 10.90	44.70 ± 11.28
Weight (kg)	76.60 ± 10.55	78.51 ± 9.48
Height (cm)	171.65 ± 6.10	169.98 ± 4.50
ASA I/II	50/20	55/15
Baseline heart rate (bpm)	76.6 ± 10.29*	81.75 ± 13.16
Baseline mean arterial pressure (mmHg)	99.07 ± 11.10*	93.44 ± 15.28
Type of surgery		
Knee arthroscopy	67	66
Femoral fractures	3	4

Data was expressed as mean ± SD or n. *P <0.05, compared with the sea level group

**Table 2:** Anaesthetic characteristics in groups

	Moderately high altitude group	Sea level group	*P* value
	(*n* = 70)	(*n* = 70)	
Duration of the block procedure (minutes)	20.38 ± 1.46	14.34 ± 0.88	*P* < 0.001
Duration of surgery (minutes)	52.15 ± 20.25	51.68 ± 15.19	0.876
Motor block time (minutes)	200.4 ± 103.2	310.2 ± 104.2	*P* < 0.05
Sensory block time (minutes)	155.2 ± 60.7	200.2 ± 50	*P* < 0.05
The application time of anaesthetic technique (minutes)	5.85 ± 0.75	5.90 ± 1.65	*P* > 0.05
The number of patients required ephedrine (n, %)	3, 4.2 %	8, 11.4 %	*P* < 0.05
PDPH incidence (n, %)	5, 7.14 %	2, 2.85 %	*P* < 0.05

Results were expressed as mean ± SD

**Table 3:** Mean spread of the sensory block of the operative side at timed intervals following local anaesthetic injection in groups

	Moderately high altitude group	Sea level group
	(*n* = 70)	(*n* = 70)
15th min	T_10_ (T_4_-T_12_)*	T_8_ (T_4_-T_12_)
30th min	T_10_ (T_4_-T_12_)*	T_8_ (T_5_-T_12_)
45th min	T_11_ (T_4_-T_12_)*	T_9_ (T_6_-T_12_)
60th min	T_12_ (T_6_-L_4_)*	T_11_ (T_6_-L_2_)
75th min	L_1_ (T_7_-S_2_)*	T_12_ (T_6_-L_3_)
90th min	L_2_ (T_7_-S_4_)*	T_12_ (T_6_-L_5_)
120th min	L_4_ (T_10_-S_4_)*	L_2_ (T_7_-S_2_)
150th min	S_1_ (T_10_-S_4_)**	L_5_ (T_2_-S_4_)

*P < 0.001, **P = 0.026, compared to the sea level group. Data were expressed as median (range)

**Table 4:** Mean bilateral motor block intensity (Bromage scale: 0–3) at timed intervals following local anaesthetic injection in groups

	Moderately high altitude group	Sea level group
	(*n* = 70)	(*n* = 70)
15th min	2.8 ± 0.52	2.70 ± 0.57
30th min	2.72 ± 0.61	2.78 ± 0.50
45th min	2.40 ± 0.82**	2.78 ± 0.50
60th min	1.72 ± 0.96*	2.58 ± 0.62
75th min	1.11 ± 0.97*	2.21 ± 0.75
90th min	0.71 ± 0.91*	1.70 ± 0.89
120th min	0.31 ± 0.80*	0.85 ± 0.82
150th min	0.22 ± 0.74	0.45 ± 0.77

*P < 0.001, **P = 0.001, compared to the sea level group

**Fig. 2:**
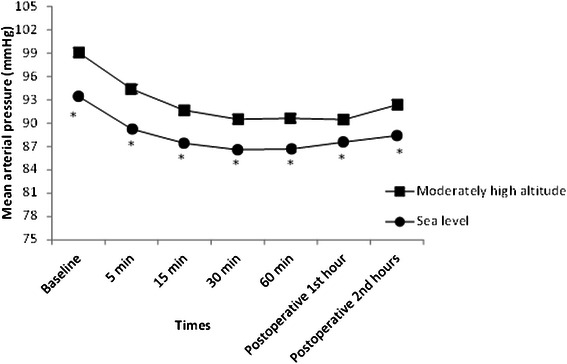
Mean arterial blood pressure values of patients in groups. *P <0.05, compared to moderately high altitude group. Patients in both groups had significantly lower MABP values during the surgery compared to baseline values (*P* < 0.05, for all)

**Fig. 3:**
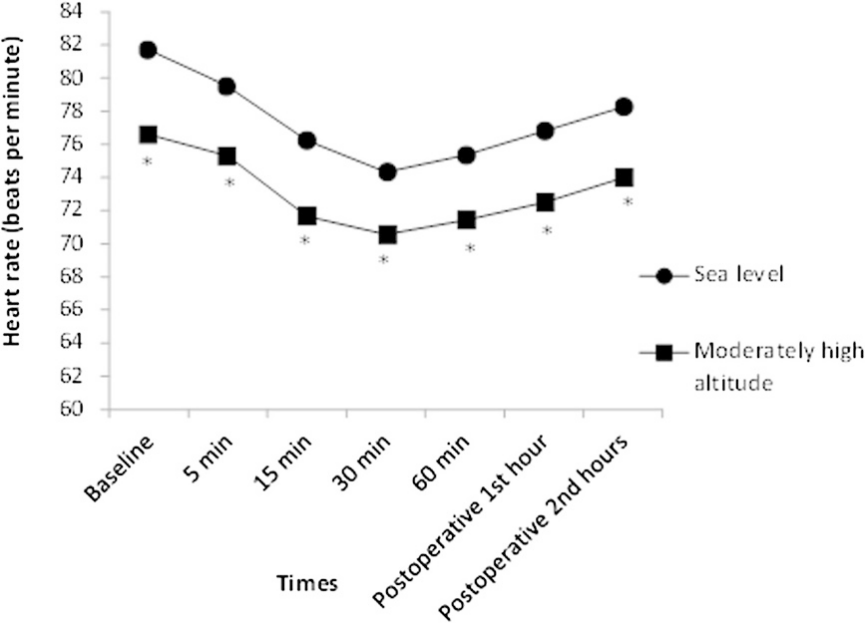
The comparison of heart rate values in groups. *P < 0.05, compared to the sea level group. Patients in both groups had significantly lower heart rate values during surgery and postoperative 1^st^ hour compared to baseline values (*P* < 0.05, for all)

Moderate altitude group had significantly higher MABP values at baseline, during surgery and in postoperative 1^st^ and 2^nd^ hours than in the sea level group (*P* < 0.05, for all). Patients in both groups had significantly lower MABP values during surgery and in postoperative 1^st^ hour compared to the baseline values (*P* < 0.005, for all) (Fig. 2). Arterial hypotension was observed in 2 patients in the sea level group and 3 patients in moderate altitude group (*P* > 0.05). Moderately high altitude group had lower heart rate values at baseline, at any time points during surgery and postoperative 1^st^ and 2^nd^ hours compared to the sea level group (*P* < 0.05). Patients in both groups had significantly lower heart rate values during surgery compared to baseline values (*P* < 0.05) (Fig. 3). Bradycardia was observed in two patients in each group. No patients in both groups had respiratory complications during surgery and post-operative period.

## Discussion

According to our literature review, this is the first study to compare onset and duration of central neuraxial block in patients living in moderately highland areas (1890 m above the sea level) with those in lowland areas (close to the sea level). Onset time of complete sensory and motor blocks was shorter in the sea level group compared to the moderately high altitude group. Also, higher upper limit of the sensory blockade and higher motor and sensorial block times were found at the sea level compared to moderately high altitude.

It has been reported that an anaesthetic technique that has the least impact on ventilation should be chosen for the patients at high altitude [1]. Also aspiration risk during anaesthesia induction increases in patients at high altitude due to the significantly delay in gastric emptying [12]. In this instance, the use of neuroaxial anaesthesia techniques at high altitude is more appropriate [1]. Spinal anaesthesia, a neuroaxial anaesthesia technique provides adequate anaesthesia for surgery with a shorter surgery onset time. Many factors affecting the intrathecal spread of injected local anaesthetics were identified such as sex, weight, height, intra-abdominal pressure and spinal anatomy [6]. Also, variability in CSF volume was reported as the most important factor that contributes to the variability in the spread of the spinal sensory anaesthesia [7]. Increasing altitude leads to the changes in cardiac, cerebral and pulmonary systems such as an increase in cerebral arterial blood flow, a decline in cardiac output and an increase in CSF volume [1–3].

We chose median effective dose of hyperbaric bupivacaine as 9 mg based upon a pilot study assessing clinical impression of motor blockade and analgesia duration in two different altitudes which this study was carried out. We observed that the sensory block spread to the T_10_ dermatome was faster in the sea level group compared to moderately high altitude group. Also, sensory block spread was significantly more extensive at the sea level than in moderately high altitude. The duration of the sensory and motor blocks were significantly shorter in moderately high altitude group than in the sea level group. These significant differences between the groups may be a result of the greater CSF volume in the moderately high altitude group as increased cerebral arterial blood flow and CSF volume has been shown to be one of the compensatory mechanisms at high altitude in response to hypoxemia [2, 3]. Although it was reported that CSF volume may have a crucial effect on intrathecal drug spread [7], there are no detailed study on this issue in the literature due to the difficulties of measuring CSF volume accurately [6]. On the other hand, cerebral blood flow alterations occur in high altitude [2] and there is an inverse correlation between the intracranial blood volume and CSF volume [13]. Also, it was reported that highlanders have lower CSF pH values than in lowlanders depending on the hyperventilation caused by hypoxia [5]. Higuchi et al. [14] reported a significant correlation between the CSF density and peak sensory block level, a positive correlation between the lumbosacral CSF volume and onset time of the complete motor block and a significant inverse correlation between the peak diastolic CSF velocity and duration of motor blockade. They concluded that CSF density and volume influence the spread of the spinal anaesthesia and that CSF volume also influences the duration of the spinal anaesthesia. It is unclear, however, as to which physiological mechanisms explain these differences in anaesthetic characteristics following spinal anaesthesia in moderately highlanders.

Heart rate values at baseline and during surgery were found to be lower in moderately highlanders compared to lowlanders in this present study. Similar to our results, Puri et al. [8] reported lower heart rate values at the baseline and during surgery in patients living at high altitude compared to patients living at low altitude. The cause of these low heart rates in moderately highlanders may be the increased parasympathetic neural activity caused chronic hypoxia at high altitude [15]. On the other hand, we observed higher MAP values at baseline and during surgery in patients living at moderately high altitude compared to the patients living at the sea level. Consistent with our results, Calbet [16] reported that chronic hypoxia causes an increase in the systemic arterial pressure in healthy humans due to the marked activation of the sympathetic nervous system and reduced tissue hypoxia associated with acclimatization.

PDPH was observed more frequently at moderate altitude in this current study. Although the exact cause of the increased incidence of PDPH at moderately high altitude remains unknown, the changes in CSF volume and pressure due to increased cerebral blood flow caused by hypoxia may be a cause for this increased incidence of PDPH at high altitude. Also, a case of headache aggravated at altitude was reported by Batsis et al. [17] They suggested that hyperventilation caused by hypoxia leads to an intracranial dehydration, cerebral vasodilation and brain engorgement. On the other hand, Wilson et al. [18] concluded that restriction in cerebral venous outflow is associated with the increased cerebral venous engorgement and with greater headache burden in response to hypoxia.

Puri et al. [8] investigated the effectiveness of the general anaesthetic agents on the anaesthetic requirements and hemodynamic variations at high and low altitudes. They concluded that high-altitude dwellers require significantly larger amounts of intravenous anaesthetic propofol. In another study, Fuzier et al. [9] evaluated the feasibility and pharmacodynamic profile of axillary brachial plexus nerve blocks performed in high altitude. They found no difference between the patients at high altitude (2877 m) and low altitude (150 m) in terms of onset times for blocks, duration of the sensory and motor blocks. However, there is no study in the literature comparing the effects of spinal anaesthesia on hemodynamic and anaesthetic parameters in different altitudes.

The limitation of this present study is that a non-invasive hemodynamic evaluation was used. The present study was focused on the anaesthetic parameters. Also, our study population was consisted of young and good health patients. So, an advanced hemodynamic monitoring was not needed. Another limittion was the relatively small patient population. On the other hand, observer bias may be a factor influencing our results.

## Conclusions

Hemodynamic variations and further anaesthetic requirements following spinal anaesthesia may be observed at moderately high altitudes compared to the sea level. Shorter onset time of complete sensory and motor blocks and higher sensory level were found in the sea level group compared to the moderately high altitude. Also, the duration of the sensory and motor blocks was longer in the sea level group than in the moderately high altitude group. These differences may be associated with a physiological adaptation to chronic hypoxia in individuals living at moderately high altitude. Further studies including a greater number of patients and reporting data on cardiac output or stroke volume variations are needed to support our findings.

## Abbreviations

CSA: Continuous spinal anaesthesia
CSF: Cerebrospinal fluid
ASA: American Society of Anaesthesiologists
PACU: Post-anaesthesia care unit
PDPH: Post-dural puncture headache
MABP: Mean arterial blood pressure
HR: Heart rate
